# Comparative efficacy of ripertamab, rituximab, and efgartigimod in chronic inflammatory demyelinating polyneuropathy: an exploratory real-world multicenter cohort study

**DOI:** 10.3389/fimmu.2026.1825584

**Published:** 2026-05-19

**Authors:** Yutong Wu, Yue Zhou, Wanshuang Yin, Xiao Zhao, Lingxu Xu, Yinchao Su, Shuxin Wang, Siyu Li, Yue Wang, Chunmei Duan, Yiliang Fang, Zhaoyou Meng

**Affiliations:** Department of Neurology, Second Affiliated Hospital of Army Medical University, Chongqing, China

**Keywords:** CIDP, cohort study, efgartigimod, ripertamab, rituximab

## Abstract

**Objective:**

To analyze and compare the efficacy of ripertamab, rituximab, and efgartigimod in patients with chronic inflammatory demyelinating polyneuropathy (CIDP), focusing on treatment response rates, relapse rates, drug safety, long-term clinical outcomes, and biomarker characteristics.

**Methods:**

A multicenter, retrospective cohort study was conducted, patients diagnosed with CIDP were enrolled if they had received at least one course of ripertamab, rituximab, or efgartigimod. A total of 60 patients were included. At baseline and at each follow-up time point, the clinical results were evaluated by disease-specific grading scale, and the correlations between biomarker spectrum and disease activity was explored.

**Results:**

A total of 60 patients were enrolled, with 27, 19, and 14 patients assigned to the rituximab, ripertamab, and efgartigimod groups, At the 3-month post-treatment assessment, the mean of the INCAT score decreased by 0.519 points in the rituximab group, 1.737 points in the ripertamab group, and 0.214 points in the efgartigimod group as compared to baseline.

**Conclusion:**

Ripertamab demonstrated efficacy in managing short-term disease outcomes in patients with CIDP, with no significant difference observed when compared to rituximab or efgartigimod. These findings suggest that ripertamab may represent a potential therapeutic strategy for CIDP in the future.

## 1 Introduction

Chronic inflammatory demyelinating polyradiculoneuropathy (CIDP) is a rare autoimmune-mediated acquired polyradiculoneuropathy, characterized by demyelination and axonal damage of the peripheral nerves (1, 2). It predominantly affects males and older adults (3), with common clinical presentations including limb weakness and sensory disturbances. The disease course, typically extending beyond 8 weeks, can be relapsing-remitting, stepwise progressive, or monophasic progressive.

The etiology of CIDP remains elusive. Evidence suggests a potential role for B cell-mediated humoral immunity. Furthermore, CD4^+^ T cells, CD8^+^ T cells, and macrophages may contribute to nerve damage under pathological conditions, potentially through mechanisms such as cytokine activation (4). Notably, a variety of peripheral neuropathy-associated antibodies, including those against gangliosides or paranodal proteins (NF155, CNTN1, and Caspr1), are known to induce axonal injury and demyelination. These antibodies play a significant role in the pathogenesis of CIDP and serve as important biomarkers for diagnosis (5).

The management of CIDP requires careful attention to the prevention of disease relapse. High-dose corticosteroid pulse therapy and intravenous immunoglobulin (IVIG) are commonly employed in the acute phase, whereas maintenance therapy with oral corticosteroids or subcutaneous immunoglobulin, followed by gradual tapering in relapse-free patients, constitutes a standard strategy (6). However, not all patients respond adequately to these interventions. One study indicated that, compared to methylprednisolone, IVIG treatment was associated with a lower probability of discontinuation due to inefficacy, intolerance, or serious adverse events within six months; nevertheless, the IVIG group exhibited a higher rate of disease worsening requiring further treatment (7). Consequently, there is an imperative need for more precise and effective therapies for patient refractory to conventional treatments.

As previously mentioned, autoantibodies against peripheral nerve components are critically involved in the pathogenesis of CIDP. The neonatal Fc receptor (FcRn) is a key receptor for the IgG Fc fragment, and inhibition of FcRn function can effectively accelerate the degradation of pathogenic IgG antibodies (8, 9). Efgartigimod, the first FcRn antagonist approved for clinical use, is a human IgG1-derived Fc fragment that competitively binds to FcRn, thereby reducing circulating IgG levels (10). A landmark large-scale randomized controlled trial demonstrated that subcutaneous efgartigimod significantly reduced the risk of relapse in treatment-responsive CIDP patients, with relapse rates of 27.9% in the efgartigimod group compared to 53.6% in the placebo group (11). Based on its efficacy, efgartigimod has now received approval for the treatment of both myasthenia gravis (MG) and CIDP.

Given the established role of B cells in the pathogenesis of CIDP, B-cell-depleting agents represent a significant therapeutic avenue. Rituximab, a chimeric anti-CD20 monoclonal antibody initially developed for lymphoma, has been explored as an alternative option for CIDP patient refractory to conventional therapies (12). Nevertheless, a randomized controlled trial demonstrated that adding rituximab followed by withdrawal of immunoglobulin therapy was not superior to immunoglobulin maintenance alone in preventing clinical relapse (13). Ripertamab is a novel human-murine chimeric anti-CD20 antibody developed based on rituximab. While structurally similar, it differs by a single amino acid substitution (valine in ripertamab vs. alanine in rituximab) at position 219 within the CH1 domain. Although ripertamab is expected to share a comparable therapeutic profile with rituximab, its application in CIDP remains unexplored. Real-world clinical data are needed to evaluate its efficacy and safety in this specific patient population.

In this study, we evaluated a cohort of 60 patients with CIDP who were treated with efgartigimod, rituximab, or ripertamab, with a mean follow-up duration of over one year. Our objectives were to analyze and compare the treatment response rates and relapse rates among the three therapeutic agents, investigate their safety profiles, and observe long-term clinical outcomes along with biomarker characteristics in the target patient population, thereby providing evidence to guide clinical application.

## 2 Methods

### 2.1 Ethical approval

This study was conducted in accordance with the principles of the Declaration of Helsinki, utilized patient data stored in the multicentric neuroimmunological disease database led by the Second Affiliated Hospital of the Army Medical University. The database has received ethical approval, with the approval number being Research 377-01. Furthermore, was approved by the Ethics Committee of The Second Affiliated Hospital of Army Medical University (Approval No.: 2024-347-02). Detailed information regarding the disease and the investigated therapeutic agents was provided to all patients and their relatives. Written informed consent was obtained from each patient or their legal guardian.

### 2.2 Study design and patients

This study is a multicenter, retrospective cohort study utilizing real-world case data. Its objective is to preliminarily explore the therapeutic effect of ripertamab in CIDP by analyzing clinical case data collected prior to the initiation of a randomized controlled trial, thereby providing clinical practice-based support for the ongoing phase III study (NCT06858722). We utilized patient data stored in the multicentric neuroimmunological disease database led by the Second Affiliated Hospital of the Army Medical University between January 2021 and September 2025, who were treated with efgartigimod, rituximab, or ripertamab. The inclusion criteria were as follows: (1) meeting the 2021 European Academy of Neurology/Peripheral Nerve Society (EAN/PNS) diagnostic criteria for CIDP (14); (2) age ≥18 years; (3) INCAT scale score of at least 1 point, with at least 1 point attributable solely to lower limb disability; (4) prior inadequate response or disease relapse following first-line immunotherapy, which included pulse corticosteroid therapy (initial infusion of 1000 mg methylprednisolone followed by tapering) or IVIg therapy (0.4 g/kg for 5 days), before initiation of the aforementioned three drugs.

The exclusion criteria comprised: (1) presence of other immune-related peripheral neuropathies, including monoclonal gammopathy of undetermined significance (MGUS) of IgG or IgA type, and polyneuropathy, organomegaly, endocrinopathy, M-protein, skin changes (POEMS) syndrome; (2) concurrent severe bacterial or viral infections, such as acquired immunodeficiency syndrome, neurosyphilis, or viral encephalitis; (3) a history of malignant tumors or organ transplantation.

### 2.3 Treatment

Among the 60 enrolled CIDP patients, 27 were treated with rituximab, 19 received ripertamab, and 14 were administered efgartigimod. Patients were numbered from 1 to 60 according to their treatment group: numbers 1–27 received rituximab, 28–46 received ripertamab, and 47–60 received efgartigimod. All patients received the standard dosage of the respective drug: 375 mg/m² for both rituximab and ripertamab, and 10 mg/kg for efgartigimod. For rituximab and ripertamab, the decision to administer subsequent infusions was based on the patient’s clinical condition, with a minimum interval of six months between courses. Efgartigimod was administered once weekly for four consecutive weeks as one treatment cycle. No patient in this study received a second cycle of efgartigimod.

### 2.4 Clinical outcome assessment

Clinical assessments were conducted at baseline, 1 month, and 3 months after treatment initiation. The following outcome measures were collected: the INCAT disability scale, the inflammatory Rasch-built Overall Disability Scale (I-RODS), and the Medical Research Council (MRC) sum score. The 3-month time point after treatment initiation was designated as the primary endpoint for analysis. The following definitions were applied: (1) Disease relapse: An increase in the INCAT score of ≥1 point compared to baseline. (2) Clinical improvement: A decrease in the INCAT score of ≥1 point compared to baseline. (3) Disease progression: A decrease in the I-RODS score of ≥4 points compared to baseline. (4) Functional improvement: An increase in the I-RODS score of ≥4 points compared to baseline.

The number and proportion of patients in each treatment group who met the criteria for disease relapse, clinical improvement, disease progression, or functional improvement at the primary endpoint (3 months) were recorded. Following the initial 3-month period, patients continued to be followed for long-term assessment. Evaluations using the same scales were performed at 6 months after the first dose and subsequently at 6-month intervals to assess the long-term efficacy and safety of the treatments.

We also collected electromyography-related data at baseline and the 3-month follow-up for analysis, including latency, amplitude, and conduction velocity for the median, ulnar, common peroneal, tibial, superficial peroneal, and sural nerves.

### 2.5 Safety assessment

Adverse events (AEs) following drug administration, including but not limited to fever and infections, were recorded for all patients in the cohort. Laboratory monitoring of immunoglobulin levels and T- and B-lymphocyte subsets was performed at baseline (prior to the initial dose) and at the 3-month time point after treatment initiation.

### 2.6 Data collection and statistical analysis

Demographic characteristics, treatment information, and laboratory data were obtained from patient medical records and follow-up visits. All data corresponding to each scheduled follow-up time point were recorded. The collected variables included gender, age, disease duration, Disease subtypes and the status of peripheral neuropathy-associated antibodies were also documented.

To expand the understanding of potential factors associated with disease outcomes in CIDP, we performed exploratory analyses by calculating certain composite indicators proposed in the NHANES database using data collected in this study, in addition to conventional variables. Based on preliminary findings from earlier small-scale studies and an initial screening of variables in this study, the residual cholesterol inflammation index (RCII) was preliminarily considered to potentially correlate with CIDP disease outcomes. Chi-square analysis was used to evaluate the effects of drug type and RCII on the four disease outcomes in CIDP. The values for each composite index were ranked from highest to lowest. Using the first quartile (Q1) and the third quartile (Q3) as cut-off points, the values were categorized into “normal(Q1-Q3)” or “abnormal (Other)” levels for subsequent analysis.

Categorical data are presented as frequencies and percentages. Continuous data are expressed as the mean and standard deviation (SD). All statistical analyses were performed using SPSS 26.0 and GraphPad Prism 9. Data standardization was based on the trends observed in laboratory parameters from baseline to 3 months after the initial treatment. Detailed criteria for all raw data and calculation methods for composite indices are provided in the supplementary tables (**Supplementary Table S1**).

## 3 Results

### 3.1 Baseline patient characteristics

The demographic and clinical characteristics of the patients are summarized in **Table 1**. All 60 patients were included in the analysis, comprising 29 males and 31 females, with a mean age of 52.18 years and a mean disease duration of 30 months. The mean baseline scores were as follows: INCAT score, 3.37; I-RODS score, 31.35; and MRC score, 51.30. Detailed scoring data for all patients are provided in supplementary (**Supplementary Table S2**).

**Table 1:** The demographic and clinical characteristics of the patients.

	Rituximab group A (n=27)	Ripertamab group B (n=19)	Efgartigimod group C (n=14)	Total(n=60)	P value A vs B A vs C B vs C
Age(years), means ± SD	47.96 ± 18.51	57.47 ± 10.92	53.14 ± 18.56	52.18 ± 16.75	0.035^a^ 0.404 0.445
Sex (female, %)	13(48.15%)	9(47.37%)	9(64.29%)	31(51.67%)	0.958 0.326 0.335
Duration (months, means ± SD)	37.15 ± 32.47	31.72 ± 27.35	34.71 ± 40.69	30.00 ± 32.71	0.543 0.848 0.814
Clinical Type (classical, %)	25(92.59%)	18(94.74%)	14(100.00%)	57(95.00%)	0.771 0.296 0.384
Peripheral neuropathy antibody (Positive, %)	17(62.96%)	11(57.89%)	8(57.14%)	36(60.00%)	0.729 0.717 0.966
INCAT Score(mean ± SD)	3.22 ± 1.67	4.26 ± 2.68	2.43 ± 1.34	3.37 ± 2.07	0.145 0.110 0.016^b^
I-RODS Score(mean ± SD)	31.48 ± 9.68	27.11 ± 13.62	36.86 ± 8.22	31.35 ± 11.21	0.239 0.071 0.016^c^
MRC Score(mean ± SD)	51.19 ± 6.31	49.68 ± 7.26	53.71 ± 5.76	51.30 ± 6.57	0.469 0.209 0.086
Limb weakness (yes, %)	22(81.48%)	18(94.74%)	10(71.43%)	50(83.33%)	0.189 0.461 0.065
Sensory abnormality (yes, %)	24(88.89%)	16(84.21%)	13(92.86%)	53(88.33%)	0.643 0.685 0.452

^a^ In terms of baseline age data, there is a significant difference between the Rituximab Group A and the Ripertamab Group B (P = 0.035).

^b^ Regarding the baseline INCAT score, there is a significant difference between the Ripertamab Group B and the Efgartigimod Group C (P = 0.016).

^c^ Regarding the baseline I-RODS score, there is a significant difference between the Ripertamab Group B and the Efgartigimod Group C (P = 0.016).

Baseline demographic and clinical characteristics of CIDP patients treated with Rituximab, Ripertamab, and Efgartigimod are presented here.

**Figure 1:**
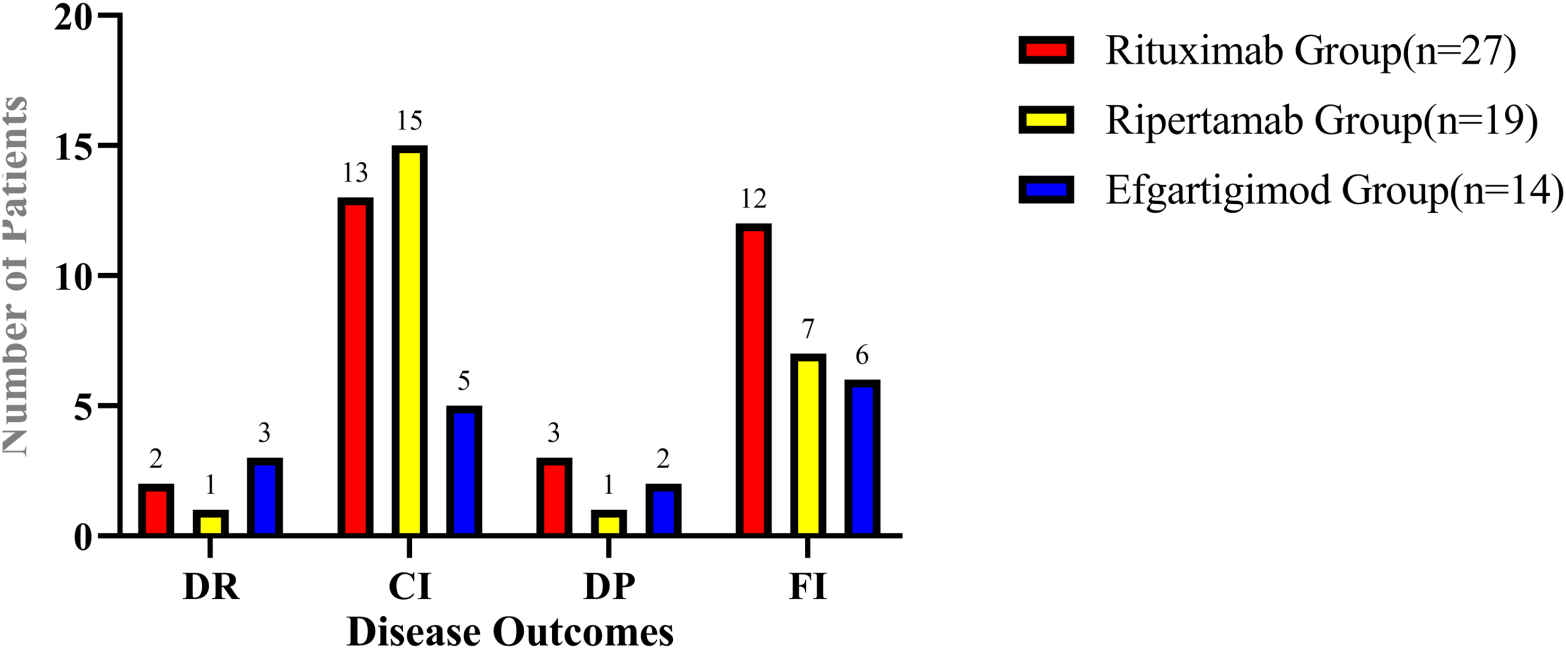
The specific number of patients in each treatment group who reached the four clinical outcomes.

According to the pre-defined criteria, disease relapse occurred in 6 patients (10.00%), clinical improvement was observed in 33 patients (55.00%), disease progression was noted in 6 patients (10.00%), functional improvement was achieved in 16 patients (45.7%). The number of patients in each group who reached the four disease outcomes is shown in **Figure 1**.

Using the chi-square test to analyze the performance of CIDP patients across three different medication groups in four distinct disease outcomes, it was found that there were no significant differences among the groups in terms of disease recurrence (P = 0.258), clinical improvement (P = 0.672), disease progression (P = 0.258), or functional improvement (P = 0.990). This indicates that compared to rituximab and efgartigimod, no significant difference in efficacy was observed for ripertamab in the treatment of CIDP.

Furthermore, the statistical analysis results in our study validated the association between RCII and the disease outcome of recurrence in CIDP. Among the 60 patients, all who experienced disease recurrence (6/6, 100%) were from the RCII abnormal group (defined as those whose calculated RCII value at the 3-month follow-up fell within the top 25% or bottom 25% of the entire cohort), while the RCII normal group (whose calculated RCII value at the 3-month follow-up was within the 25th to 75th percentile of the cohort) appeared to completely avoid the risk of reaching this outcome (0/30, 0%). Results are illustrated in **Table 2**. The occurrence of such extreme results may suggest that RCII holds potential monitoring value for CIDP disease activity risk. However, similarly, the retrospective nature of the study and the small sample size introduce a significant risk of overfitting, which severely limits the reliability of the conclusions. Given that existing research has demonstrated a close relationship between the disease progression of CIDP and lipid metabolism (15), large-scale prospective studies are required to clarify whether RCII truly holds important value in predicting CIDP prognosis.

**Table 2:** The association between the RCII index and disease recurrence in patients.

	Relapse	No relapse	Total
Normal RCII group	0	30	30
Abnormal RCII group	6	24	30
P		<0.001	

All patients in the normal RCII group (100%, 30/30) exhibited no relapse, while all 6 relapsed cases occurred in the abnormal RCII group.

### 3.2 Electromyography

Analysis of electromyography data from 41 patients at baseline and the 3-month follow-up showed that all patient groups achieved a certain degree of improvement after treatment. The overall analysis revealed that, in motor nerve conduction, the latency, amplitude, and conduction velocity of the median nerve; the amplitude of the ulnar nerve; the latency, amplitude, and conduction velocity of the common peroneal nerve; and the amplitude of the tibial nerve showed varying degrees of improvement at follow-up. In sensory nerve conduction, the conduction velocity of the median nerve, the amplitude of the ulnar nerve, the amplitude and conduction velocity of the superficial peroneal nerve, and the amplitude and conduction velocity of the sural nerve were also found to have improved at follow-up. Details are shown in **Table 3**.

**Table 3:** Changes in EMG related indicators before and after patient treatment.

	Rituximab group(n=21)		Ripertamab group(n=13)		Efgartigimod group(n=7)		Total(n=41)	
	Baseline	3 months	Baseline	3 months	Baseline	3 months	Baseline	3 months
Motor nerve conduction velocity								
Median Nerve								
Latent Period	10.2 ± 6.78	9.66 ± 4.36	10.99 ± 4.12	9.43 ± 3.95	9.56 ± 4.71	11.48 ± 7.25	10.34 ± 5.62	9.90 ± 4.76
Amplitude	3.51 ± 3.01	4.12 ± 2.67	4.14 ± 2.35	4.87 ± 2.59	6.29 ± 4.74	7.32 ± 6.21	4.18 ± 3.25	4.91 ± 3.57
Conduction Velocity	36 ± 19.54	46.46 ± 14.86	41.10 ± 12.68	45.02 ± 12.85	44.63 ± 13.96	50.03 ± 15.04	39.09 ± 16.75	46.61 ± 14.03
Ulnar nerve								
Latent Period	8.03 ± 5.56	8.13 ± 4.87	8.37 ± 3.68	8.24 ± 3.66	8.11 ± 4.72	8.66 ± 5.14	8.15 ± 4.79	8.25 ± 4.46
Amplitude	3.32 ± 1.82	4.25 ± 2.19	3.69 ± 2.10	6.46 ± 4.16	3.58 ± 2.92	7.28 ± 6.04	3.48 ± 2.07	5.47 ± 3.84
Conduction Velocity	54.18 ± 6.75	51.90 ± 13.49	51.74 ± 11.45	51.48 ± 12.69	52.31 ± 17.16	53.09 ± 16.61	53.09 ± 10.37	51.97 ± 13.45
Common peroneal nerve								
Latent Period	11.76 ± 7.06	9.31 ± 5.00	11.15 ± 5.88	9.01 ± 6.52	12.54 ± 5.00	10.44 ± 13.39	11.70 ± 6.26	9.41 ± 7.24
Amplitude	1.68 ± 1.55	2.09 ± 2.11	1.45 ± 1.27	5.62 ± 4.66	2.49 ± 2.66	8.60 ± 7.25	1.75 ± 1.70	4.32 ± 4.80
Conduction Velocity	31.73 ± 16.55	38.05 ± 18.12	32.23 ± 15.97	42.76 ± 18.03	39.89 ± 12.24	39.57 ± 24.31	33.28 ± 15.66	39.80 ± 18.84
Tibial nerve								
Latent Period	13.61 ± 3.92	12.80 ± 3.39	13.03 ± 4.28	7.35 ± 4.64	10.96 ± 1.58	14.94 ± 9.68	12.98 ± 3.81	11.44 ± 5.90
Amplitude	3.66 ± 2.68	3.54 ± 2.64	2.91 ± 2.68	6.01 ± 5.08	4.58 ± 2.40	6.14 ± 3.00	3.58 ± 2.63	4.77 ± 3.77
Conduction Velocity	43.8 ± 12.72	47.80 ± 16.43	44.58 ± 7.66	40.67 ± 24.81	49.67 ± 3.29	48.07 ± 15.57	45.05 ± 10.24	45.59 ± 19.17
Sensory nerve conduction velocity								
Median Nerve								
Latent Period	2.28 ± 1.36	2.27 ± 1.05	2.32 ± 1.114	3.38 ± 1.80	2.35 ± 1.36	4.52 ± 3.08	2.31 ± 1.26	3.01 ± 1.92
Amplitude	8.52 ± 11.53	8.91 ± 7.58	5.26 ± 4.67	7.13 ± 4.92	16.69 ± 19.75	7.08 ± 6.25	8.88 ± 12.10	8.03 ± 6.53
Conduction Velocity	38.72 ± 21.33	48.34 ± 17.57	37.77 ± 18.07	51.23 ± 18.50	46.03 ± 24.35	50.84 ± 23.35	39.67 ± 20.57	49.68 ± 18.46
Ulnar nerve								
Latent Period	2.09 ± 0.72	2.19 ± 1.26	2.27 ± 0.92	4.25 ± 3.05	2.00 ± 1.16	4.59 ± 3.98	2.13 ± 0.85	3.25 ± 2.68
Amplitude	6.99 ± 4.67	5.95 ± 4.39	4.74 ± 3.69	7.10 ± 6.14	6.56 ± 5.95	7.27 ± 6.29	6.21 ± 4.62	6.54 ± 5.22
Conduction Velocity	51.26 ± 15.86	49.49 ± 19.02	45.38 ± 15.23	47.23 ± 21.65	51.33 ± 26.39	36.19 ± 25.16	49.40 ± 17.54	46.50 ± 20.97
Superficial peroneal nerve								
Latent Period	2.05 ± 1.17	2.07 ± 1.04	1.34 ± 1.41	3.03 ± 2.73	1.35 ± 1.31	5.79 ± 2.88	1.71 ± 1.29	3.01 ± 2.42
Amplitude	6.10 ± 7.24	7.88 ± 6.32	3.41 ± 5.24	5.36 ± 4.20	4.80 ± 6.27	11.79 ± 6.45	5.02 ± 6.47	7.75 ± 6.02
Conduction Velocity	37.06 ± 20.78	44.64 ± 20.11	25.26 ± 25.20	41.25 ± 28.82	28.16 ± 26.66	50.86 ± 11.22	31.80 ± 23.32	44.63 ± 21.93
Sural nerve								
Latent Period	2.00 ± 1.04	2.47 ± 1.18	2.04 ± 1.03	4.09 ± 1.79	1.23 ± 1.04	5.48 ± 2.20	1.88 ± 1.05	3.50 ± 1.93
Amplitude	3.76 ± 2.67	7.22 ± 4.19	5.18 ± 3.12	7.19 ± 6.15	3.24 ± 2.34	12.46 ± 5.47	4.12 ± 2.81	8.11 ± 5.35
Conduction Velocity	38.04 ± 13.28	54.09 ± 13.89	43.71 ± 6.68	55.08 ± 6.06	34.17 ± 16.10	49.83 ± 10.23	39.18 ± 12.33	53.68 ± 11.25

### 3.3 Long-term efficacy analysis

**Figure 2:**
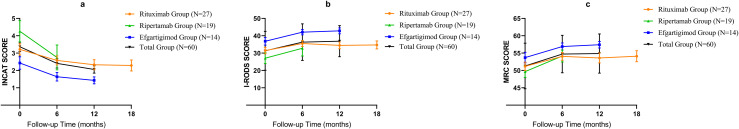
Comparison of curative effect scores of different drugs.

The overall trends in these scores across groups are illustrated in **Figure 2**. Data analysis revealed no significant differences in the 18-month improvements among the three patient groups treated with rituximab, efgartigimod, and ripertamab, respectively. However, follow-up results beyond 18 months were excluded because the number of patients who reached this time point did not meet 50% of the group’s total, thus preventing the drawing of longer-term conclusions from the existing findings. Further research is needed to validate these results.

### 3.4 Adverse events

Among the 60 patients, 11 experienced fever during drug infusion: 5 in the rituximab group, 3 in the ripertamab group, and 3 in the efgartigimod group. One patient developed a rash after infusion of ripertamab. No serious adverse events (SAEs) were reported. The incidence of adverse events for each patient group in this study has been summarized in **Table 4**.

**Table 4:** Adverse events for patient groups.

	Rituximab group(n=27)	Ripertamab group(n=19)	Efgartigimod group(n=14)	Total(n=60)
Adverse Events				
Fever, %	5(18.52%)	3(15.79%)	3(21.43%)	11(18.33%)
Rash, %	0	1(5.26%)	0	1(1.67%)
Serious Adverse Events, %	0	0	0	0

### 3.5 Analysis of safety biomarkers

Serum immunoglobulin levels and T and B-lymphocyte subset monitoring data were collected at baseline and 3 months after medication to assess the immunomodulatory effects of the related drug.

As shown in **Table 5**, immunoglobulin levels measured at baseline and at the 3-month follow-up decreased in all patient groups. In terms of the overall values, IgG decreased by 22.69%, IgA by 25.22%, IgM by 22.40%, and IgE by 48.91%.

**Table 5:** Changes in patient immunoglobulin levels.

	Rituximab group(n=19)		Ripertamab group(n=18)		Efgartigimod group(n=10)		Total(n=47)	
	Baseline	3 months	Baseline	3 months	Baseline	3 months	Baseline	3 months
IgG(mean ± SD)	11.11 ± 3.57	9.2 ± 3.66	13.71 ± 7.03	11.17 ± 8.41	10.74 ± 5.79	6.11 ± 2.56	12.03 ± 5.62	9.3 ± 6.02
IgA(mean ± SD)	2.29 ± 1.07	1.79 ± 0.99	2.32 ± 1.59	1.55 ± 0.68	2.1 ± 1.55	1.74 ± 1.43	2.26 ± 1.37	1.69 ± 0.99
IgM(mean ± SD)	1.07 ± 0.50	0.85 ± 0.47	1.59 ± 0.9	1.21 ± 0.95	1.00 ± 0.47	0.77 ± 0.46	1.25 ± 0.72	0.97 ± 0.71
IgE(mean ± SD)	86.15 ± 116.59	45.15 ± 53.59	260.36 ± 646.32	113.85 ± 202.9	87.56 ± 111.91	77.07 ± 159.67	153.17 ± 411.62	78.25 ± 149.25

The mean serum levels of IgG, IgA, IgM and IgE decreased from baseline.

Analysis of peripheral T-cell subsets in 31 patients (rituximab group, 14; ripertamab group, 11; Efgartigimod Group, 6) revealed distinct patterns. In total, the percentage of total T-cells, CD4+ T cells and CD8^+^ T cells decreased at 3 months, the three decreased by 4.07%, 2.01%, and 7.57%, respectively. In ripertamab group, the percentage of CD4^+^ T cells showed increases, the proportion of T-cell subsets in efgartigimod group showed a slight increase (**Table 6**).

**Table 6:** Changes in patient T cells levels.

	Rituximab group(n=14)		Ripertamab group(n=11)		Efgartigimod group(n=6)		Total(n=31)		
	Baseline	3 months	Baseline	3 months	Baseline	3 months	Baseline		3 months
Total T Cells (%, mean ± SD)	77.83 ± 10.88	71.69 ± 8.47	81.11 ± 9.68	79.82 ± 8.41	68.11 ± 14.29	68.58 ± 12.42	77.11 ± 11.79	73.97 ± 10.06	
CD4^+^T Cells (%, mean ± SD)	40.95 ± 7.12	38.19 ± 9.52	44.6 ± 8.76	46.48 ± 7.6	38.11 ± 4.1	36.72 ± 3.64	41.69 ± 7.5	40.85 ± 8.9	
CD8^+^ T Cells (%, mean ± SD)	32.47 ± 8.94	28.72 ± 8.24	32.66 ± 10.58	30.71 ± 10.91	25.52 ± 12.1	25.66 ± 14.87	31.19± 10.21	28.83 ± 10.45	

In the rituximab group, the percentage of total T-cell counts, CD8+ T cells and CD4+ T cells decreased at 3 months, while in ripertamab group, the percentage of CD4+ T cells slightly increased. In summary, compared to the baseline level, the patient’s T-cell levels showed a declining trend at the 3-month follow-up.

Furthermore, analysis of B-cell subsets in 10 patients from the ripertamab group showed a significant decrease at 3 months post-treatment, including CD19^+^ B cells, CD19^+^CD20^+^ B cells, memory B cells, switch memory B cells, and plasmablasts (**Table 7**).

**Table 7:** Changes in patient B cells levels.

	Ripertamab Group (n=10)	
	Baseline	3 months
CD19^+^ B Cells (%, mean ± SD) CD19^+^ B Cells Count	10.41 ± 9.61 103.82 ± 149.17	1.62 ± 4.78 7.64 ± 18.53
CD19^+^20^+^B Cells (%, mean ± SD)	10.19 ± 9.57	1.6 ± 4.79
CD19^+^20^+^B Cells Count	98.65 ± 151.73	6.95 ± 18.37
Memory B Cells Count	33.31 ± 55.42	2.95 ± 6.47
Switch Memory B Cells Count	23.66 ± 34.05	2.16 ± 4.73
Plasmablast Count	0.72 ± 0.84	0.14 ± 0.15

## 4 Discussion

CIDP is a heterogeneous chronic neuroimmune disorder with diverse clinical presentations and phenotypic variants, posing significant challenges in diagnosis and management. In this real-world multicenter cohort study, we evaluated the outcomes of 60 patients treated with one of two CD20^+^ B-cell-depleting agents (rituximab or ripertamab) or the FcRn antagonist efgartigimod, with a minimum follow-up of 3 months and a mean follow-up exceeding one year.

The relapse rate in the efgartigimod group in our study (37.5%) was slightly higher than that reported in the prior ADHERE randomized controlled trial, suggesting a potential need for a larger sample size for more precise estimation (11). Consistent with the ADHERE trial, which indicated early clinical improvement by week 4, our observations of increased I-RODS and MRC scores in a subset of our patients suggest a prompt functional and strength recovery with efgartigimod.

Ripertamab and rituximab are both CD20^+^-targeting B-cell-depleting agents that share identical antigen-binding sites and variable region amino acid sequences. The primary structural distinction lies in a single amino acid substitution at position 219 within the CH1 region, where ripertamab contains a valine residue compared to the alanine found in rituximab. Furthermore, ripertamab has a lower murine protein content than rituximab. Previous evidence from two clinical trials and two retrospective studies has indicated that ripertamab and rituximab share comparable efficacy and safety profiles (16–19).

This study also explored the association between laboratory parameters (including novel composite indicators) and treatment efficacy in CIDP patients. Our analysis revealed a potential correlation between an increase in RCII at 3 months post-treatment and a decreased likelihood of clinical improvement. RCII is a comprehensive biomarker that integrates lipid metabolism and systemic inflammation, previously primarily used for cardiovascular risk assessment. However, the study design and sample size introduce a substantial risk of overfitting, which cannot be avoided. Therefore, this finding remains only a hypothesis and requires validation through larger-scale studies in the future to assess its value in disease monitoring.

The CIDP patients in our study demonstrated favorable tolerability to efgartigimod, rituximab, and ripertamab, with no SAEs reported. Infusion-related fever occurred in only 11 patients (5 in the rituximab group), representing an incidence lower than that reported in previous studies (20, 21). This discrepancy may be attributable to the real-world design of the present study, where mild or non-urgent adverse events might have been underreported or not systematically captured.

Regarding safety profiles, efgartigimod may potentially offer a superior safety advantage over conventional B-cell-depleting agents, as it selectively reduces IgG levels without significantly impacting other immunoglobulin classes (10). This targeted mechanism could translate to a more favorable risk-benefit ratio in certain patient populations. We must point out that it is somewhat inappropriate for this study to simply juxtapose the long-term efficacy of therapies with different pharmacokinetic profiles and durations of action (primarily referring to CD20 monoclonal antibodies and FcRn antagonists). Efgartigimod is designed primarily for rapid symptom relief, but its duration is short, requiring repeated administration; in contrast, anti-CD20 monoclonal antibodies are characterized by long-term efficacy and sustained action. In this study, the efgartigimod group received only four doses within one month, and it is difficult to attribute their long-term efficacy to benefits from the early treatment. However, it is essential not to overlook the drug’s pharmacological characteristics and to make arbitrary claims about its poor long-term efficacy. We emphasize that the observations at the long-term time point in this study reflect more the differences in real-world application patterns of various treatment strategies rather than simply differences in drug efficacy.

Our study has obvious limitations in terms of the comparability of baseline data. As shown in **Table 1**, baseline disability scores (such as INCAT and I-RODS scores) of patients in ripertamab group differed significantly from those in other groups. This discrepancy represents an important confounding factor: patients with more severe baseline disease have greater potential for improvement, which may influence the objective assessment of treatment efficacy. Due to the small sample size, we were unable to completely eliminate this influencing factor. Therefore, the observed results should be interpreted as reflecting the actual treatment effects in different patient populations rather than as adjusted comparative efficacy.

## 5 Limitations

This study has several limitations. First, its retrospective design, despite follow-ups conducted at predetermined intervals, may lead to underreporting of mild adverse events, thereby hindering an accurate safety assessment. Second, due to the retrospective nature and limited sample size of only 60 cases, statistical methods such as propensity score matching could not be effectively applied to adjust outcomes. Selection bias and information bias cannot be ruled out. Therefore, this study may be insufficient to support robust comparisons of the treatment effects of the three CIDP therapies or any conclusions regarding superiority. It primarily serves to share clinical exploratory findings and observations. Definitive efficacy conclusions require further validation through large-scale prospective studies. Finally, due to practical constraints and variations in patient compliance, data on T-cell and B-cell subsets, immunoglobulin, and complement levels were incomplete, which impeded comprehensive statistical analysis. We claimed that retrospective studies cannot replace randomized controlled trials (RCTs), they do provide important insights into the use of therapies in routine, complex clinical settings—information that is often difficult to capture in RCTs. Retrospective studies hold unique significance for guiding clinical decision-making, particularly in conditions like CIDP, where cases treated with the relevant therapies are relatively scarce.

## 6 Conclusion

Given these limitations, particularly the sample size constraints inherent in a retrospective study, the finding of this study should be interpreted with caution. Further validation through large-scale, prospective, randomized controlled trials is essential to definitively evaluate the long-term efficacy and safety of ripertamab in CIDP. Nonetheless, these preliminary findings suggest that ripertamab may hold promise as a potential alternative therapy for CIDP patients.

## Data Availability

The raw data supporting the conclusions of this article will be made available by the authors, without undue reservation.

## References

[1] Broers MC Bunschoten C Nieboer D Lingsma HF Jacobs BC . Incidence and prevalence of chronic inflammatory demyelinating polyradiculoneuropathy: a systematic review and meta-analysis. Neuroepidemiology. (2019) 52(3-4):161–172. doi: 10.1159/000494291. PMID: 30669140 PMC6518865

[2] Mathey EK Park SB Hughes RAC Pollard JD Armati PJ Barnett MH . Chronic inflammatory demyelinating polyradiculoneuropathy: from pathology to phenotype. J Neurol Neurosurg Psychiatry. (2015) 86(9):973–985. doi: 10.1136/jnnp-2014-309697. PMID: 25677463 PMC4552934

[3] Querol L Crabtree M . Systematic literature review of burden of illness in chronic inflammatory demyelinating polyneuropathy (CIDP). J Neurol. (2021) 268:3706–16. doi: 10.1007/s00415-020-09998-8. PMID: 32583051 PMC8463372

[4] Querol L Lleixà C . Novel immunological and therapeutic insights in guillain-barré syndrome and CIDP. Neurotherapeutics. (2021) 18:2222–35. doi: 10.1007/s13311-021-01117-3. PMID: 34549385 PMC8455117

[5] Querol LA Hartung HP Lewis RA Van Doorn PA Hammond TR Atassi N . The role of the complement system in chronic inflammatory demyelinating polyneuropathy: implications for complement-targeted therapies. Neurotherapeutics. (2022) 19:864–73. doi: 10.1007/s13311-022-01221-y. PMID: 35378684 PMC9294101

[6] Bunschoten C Jacobs BC Van den Bergh PYK Cornblath DR van Doorn PA . Progress in diagnosis and treatment of chronic inflammatory demyelinating polyradiculoneuropathy. Lancet Neurol. (2019) 18(8):784–794. doi: 10.1016/S1474-4422(19)30144-9. PMID: 31076244

[7] Nobile-Orazio E Cocito D Jann S Uncini A Beghi E Messina P . Intravenous immunoglobulin versus intravenous methylprednisolone for chronic inflammatory demyelinating polyradiculoneuropathy: a randomised controlled trial. Lancet Neurol. (2012) 11(6):493–502. doi: 10.1016/S1474-4422(12)70093-5. PMID: 22578914

[8] Pyzik M Kozicky LK Gandhi AK Blumberg RS . The therapeutic age of the neonatal Fc receptor. Nat Rev Immunol. (2023) 23:415–32. doi: 10.1038/s41577-022-00821-1. PMID: 36726033 PMC9891766

[9] Dalakas MC Spaeth PJ . The importance of FcRn in neuro-immunotherapies: From IgG catabolism, FCGRT gene polymorphisms, IVIg dosing and efficiency to specific FcRn inhibitors. Ther Adv Neurol Disord. (2021) 14:1756286421997381. doi: 10.1177/1756286421997381. PMID: 33717213 PMC7917847

[10] Ulrichts P Guglietta A Dreier T van Bragt T Hanssens V Hofman E . Neonatal Fc receptor antagonist efgartigimod safely and sustainably reduces IgGs in humans. J Clin Invest. (2018) 128:4372–86. doi: 10.1172/jci97911. PMID: 30040076 PMC6159959

[11] Allen JA Lin J Basta I Dysgaard T Eggers C Guptill JT . Safety, tolerability, and efficacy of subcutaneous efgartigimod in patients with chronic inflammatory demyelinating polyradiculoneuropathy (ADHERE): a multicentre, randomised-withdrawal, double-blind, placebo-controlled, phase 2 trial. Lancet Neurol. (2024) 23(10):1013–1024. doi: 10.1016/S1474-4422(24)00309-0. PMID: 39304241

[12] Roux T Debs R Maisonobe T Lenglet T Delorme C Louapre C . Rituximab in chronic inflammatory demyelinating polyradiculoneuropathy with associated diseases. J Peripher Nerv Syst. (2018) 23(4):235–240. doi: 10.1111/jns.12287. PMID: 30203907

[13] Nobile-Orazio E Cocito D Manganelli F Fazio R Lauria Pinter G Benedetti L . Rituximab versus placebo for chronic inflammatory demyelinating polyradiculoneuropathy: a randomized trial. Brain. (2025) 148(4):1112–1121. doi: 10.1093/brain/awae400. PMID: 39658326 PMC11967823

[14] Van den Bergh PYK van Doorn PA . European Academy of Neurology/Peripheral Nerve Society guideline on diagnosis and treatment of chronic inflammatory demyelinating polyradiculoneuropathy: Report of a joint Task Force-second revision. Eur J Neurol. (2021) 28:3556–83. doi: 10.1111/ene.14959. PMID: 34327760

[15] Auf dem Brinke K Borsch LM Klose C Zschüntzsch J Vinhoven L Nietert M . Plasma lipidomic patterns associated with disease activity in chronic inflammatory demyelinating polyradiculoneuropathy (LIPID-CIDP). J Lipid Res. (2025) 66(11):100903. doi: 10.1016/j.jlr.2025.100903. PMID: 40972969 PMC12593536

[16] Shi Y Zhang Q Hong X Wang Z Gao Y Zou L . Comparison of efficacy and safety of ripertamab (SCT400) versus rituximab (Mabthera®) in combination with CHOP in patients with previously untreated CD20-positive diffuse large B-cell lymphoma: a randomized, single-blind, phase III clinical trial. Hematol Oncol. (2022) 40(5):930–940. doi: 10.1002/hon.3054. PMID: 35858181

[17] Han X Zhang M Wang H Zhang Q Li W Hao M . A multi-center, open-label, randomized, parallel-controlled phase II study comparing pharmacokinetic, pharmacodynamics and safety of ripertamab (SCT400) to rituximab (MabThera®) in patients with CD20-positive B-cell non-Hodgkin lymphoma. Chin J Cancer Res. (2022) 34(6):601–611. doi: 10.21147/j.issn.1000-9604.2022.06.08. PMID: 36714342 PMC9829503

[18] Wang X Song X Sun N Chang W . Efficacy and safety of ripertamab for treating primary membranous nephropathy among adults: a multicenter, retrospective, real-world study. Front Immunol. (2025) 16:1540694. doi: 10.3389/fimmu.2025.1540694. PMID: 40207232 PMC11979284

[19] Li X Liu Y Zhu X Jin H Zhang X Wang L . The efficacy and safety of ripertamab in the treatment of idiopathic membranous nephropathy: a retrospective multicenter cohort study. Sci Rep. (2025) 15(1):20567. doi: 10.1038/s41598-025-06046-1. PMID: 40593173 PMC12216789

[20] Wang Y Ma X Yang X Bai S Zang X Liao L . A multicenter retrospective study on comparing the efficacy and safety of the therapy of intermittent cyclophosphamide and corticosteroids versus rituximab for primary membranous nephropathy. Ren Fail. (2024) 46:2409353. doi: 10.1080/0886022X.2024.2409353. PMID: 39351796 PMC11445897

[21] Fervenza F Appel G Barbour S Rovin B Lafayette R Aslam N . Rituximab or cyclosporine in the treatment of membranous nephropathy. N Engl J Med. (2019) 381:36–46. doi: 10.1056/NEJMoa1814427. PMID: 31269364

