# Supplementary material for: Comparative efficacy of ripertamab, rituximab, and efgartigimod in chronic inflammatory demyelinating polyneuropathy: an exploratory real-world multicenter cohort study
Source: Front Immunol. 2026 May 19;17:1825584. doi: 10.3389/fimmu.2026.1825584 (PMC13226162; doi:10.3389/fimmu.2026.1825584)
Supplement: Supplementary file 1 [file Table1.docx]

**Table S1. Detailed criteria for data and calculation methods for composite indices.**

| Data type | Define the range of "normal" | Define the range of "abnormal" |
| --- | --- | --- |
| Sex | Male | Female |
| Age | 18-60 | ≥61 |
| Hypertension | Yes | No |
| Coronary heart disease | Yes | No |
| Diabetes | Yes | No |
| Disease classification | Classical CIDP | Other types |
| Peripheral neuropathy antibody | Positive | Negative |
| Total cholesterol | 0-5.2mmol/L | ＞5.2mmol/L |
| High density lipoprotein cholesterol | ＞1mmol/L | ≤1mmol/L |
| Low density lipoprotein cholesterol | ≤3.4mmol/L | ＞3.4mmol/L |
| High sensitivity C-reactive protein | 0-8mg/L | ＞8mg/L |
| Fasting triglyceride | ≤1.7mmol/L | ＞1.7mmol/L |
| Cerebrospinal fluid protein | ≤0.45g/L | ＞0.45g/L |
| Cerebrospinal fluid leukocytes | ≤8×10^6^/L | ＞8×10^6^/L |
| Body mass index | 18.5-23.9 | Other |
| Percentage of neutrophils | 40-75% | Other |
| Blood platelet | 100-300×10^9^/L | Other |
| Percentage of monocytes | 3-10~~%~~ | Other |
| Lymphocyte percentage | 20-50% | Other |
| Fasting blood-glucose | 3.9-6.1mmol/L | Other |
| Albumin | 40-55g/L | Other |
| Neutrophil count | 1.8-6.3×10^9^/L | Other |
| Monocyte count | 0.1-0.6×10^9^/L | Other |
| Lymphocyte count | 1.1-3.2×10^9^/L | Other |
| Disease relapse | No | Yes |
| Clinical improvement | Yes | No |
| Disease progression | No | Yes |
| Functional improvement | Yes | No |
| course of a disease | 3-12 months | ＞12months |
| The length of hospitalization | ≤15 days | ＞15days |
| Times of hospitalization | ≤2 | ＞3 |
| Types of medication | Ripertamab | Rituximab or efgartigimod |

Synthetic index:

Aggregate Index of Systemic Inflammation(AISI): Neutrophil count × platelet count × monocyte count

Residual cholesterol inflammation index (RCII): (total cholesterol-high density lipoprotein cholesterol-low density lipoprotein cholesterol) × high sensitivity C-reactive protein ÷10

C-reactive protein-triglyceride glucose index (CTI): Ln (high sensitivity C-reactive protein) +Ln (fasting triglyceride × fasting blood glucose)

Systemic immune inflammatory index (SII): platelet count × neutrophil count/lymphocyte count

Lymphocyte to high density lipoprotein ratio (LHR): lymphocyte count/high density lipoprotein cholesterol

Monocyte to HDL ratio (MHR): monocyte count/high density lipoprotein cholesterol

Plasma atherosclerosis index (AIP): LG (fasting triglyceride/high density lipoprotein cholesterol)

Neutrophil to albumin ratio (NPAR): neutrophil percentage ×100/ albumin

CALLY index (CRP-albumin-lyric index): albumin × lymphocyte count/high sensitivity C-reactive protein

Inflammation burden index (IBI): high-sensitivity C-reactive protein × neutrophil count/lymphocyte count.

Using the first quartile (Q1) and the third quartile (Q3) as cut-off points, the values were categorized into "normal(Q1-Q3)" or "abnormal (Other)" levels for subsequent analysis.

Sex：male=1 female=2

Age: 19-35=1 36-60=2 ≥61=3

Duration:6-12=1 13-24=2 25-60=3 ≥61=4

Clinical Type: classical=1 other=2

Baseline INCAT Score: 0-2=1 3-4=2 5-6=3 7-8=4 9-10=5

Baseline MRC Score:55-60=1 50-54=2 ≤49=3

Baseline I-RODS Score:40-48=1 30-39=2 20-29=3 ≤19=4

Limb weakness: Yes=1 No=2

Sensory abnormality: Yes=1 No=2
